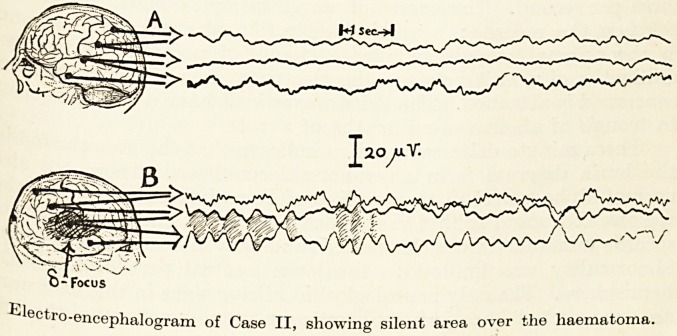# Traumatic Extradural Hæmorrhage. Part II

**Published:** 1940

**Authors:** W. Grey Walter

**Affiliations:** Physiologist to the Burden Neurological Institute, Bristol


### Part II.

### THE CLINICAL APPLICATION OF ELECTRO-ENCEPHALOGRAPHY.

BY

W. Grey Walter, M.A.

*Physiologist to the Burden Neurological Institute, Bristol.*

In a recent article in this *Journal** an account was given of the historical development and present practical utility of electro-encephalography. Attention was confined to the general clinical aspects of the new science since the technical details are difficult to expound without reference to particular cases and actual records obtained with the apparatus. However, in Case II, recorded above, location of the lesion by electro-encephalography proved more accurate than the conventional clinical methods, and led to a successful operation. It was thought that this case, being of local as well as surgical interest, might provide a nucleus for a description of the means whereby such location is achieved.

Like electro-cardiography, electro-encephalography involves registration of the minute electrical currents produced by active living cells. Examination of these " bio-electric " phenomena gives us the best " inside information " about the course of living processes which we can obtain at present, but there are many technical difficulties. The first is the small size of the currents. They bear the same sort of relation to those used, say, in an electric flashlight

* Walter, W. Grey, *Bristol Med. Chir. Jour.*, 1939, lvii. 215.

as the size of a red blood cell does to that of the human body. They must be enormously magnified or "amplified" before they can be seen or recorded. The second, more abstruse, difficulty is that the existence of an electric current depends upon a difference of electrical pressure between two places, and what the modern electrical instruments measure is that *difference of pressure* or electrical "potential." This is not an absolute sort of measurement: the potential of one point is measured with respect to another and this means that, when a potential difference is discovered, no single measurement can determine whether it is due to a rise of potential at one of the points or a fall at the other.

In the case of the heart this ambiguity is of no great practical importance since it is known that the electro-cardiogram is due to the potentials from the heart muscle and the location of the heart is already known; moreover, the potential pattern in the familiar P Q R S T waves is regularly repeated so that leads can be taken successively from the conventional three or four positions without fear that the form of the waves has altered while the connections were being changed.

But in the brain we have an enormously complex organ, any part of which may produce a series of electrical waves, and these waves are never the same for long in size or shape. If we merely place one lead on one side of the head and one on the other and pick up a fluctuating electrical potential between the two we shall still not know which of the leads or electrodes is nearer to the part of the brain generating the potentials. If we attempt to check this by moving the electrodes to another position we cannot be certain that the region of activity has not also shifted meanwhile. The only way to get around this difficulty is to take records from several points simultaneously so that each individual wave may be tracked down and located. That is why in the records in the Figure three wavy lines are seen. Each line represents the momentary fluctuations

Electro-encephalogram of Case II, showing silent area over the haematoma.

of potential between two electrodes represented on the head diagram as black dots. The recorders are so connected that, when two adjacent lines are seen to approach or recede from one another, the region of activity may be inferred to be near the electrode common to both : when the lines rise and fall together the focus of activity is towards one end or the other of the electrode chain. When the record shows waves rising and falling in opposition to one another the waves are said to be "out of phase"; they are "in phase" when they rise and fall together.

Now in Figure A, although there is a certain amount of irregularity in the record, there is very little in the way of rhythmic activity. This record is normal for a person in the alert state. Towards the end of the Record A, the bottom line shows small ripples; these are the so-called "Alpha" waves found in the parieto-occipital region in normal subjects, and indicating a state of relative physiological rest in this area. At this point it would be well to emphasize that, in the brain, rhythmic *electrical* activity is seen chiefly when there is a *decrease* in functional activity. This is believed to be due to the fact that when the brain is performing some normal function, comparatively few of the neurones are doing the same thing; when the nerve cells are released from activity they "mark time" together and the sum of their activities is large enough to be detectable on the outside of the head. Moreover, when some pathological process prevents their functioning they will again tend to fall into step, but at a slower rate, a sort of funeral march. These slow pathognomonic waves are called "Delta waves." They are clearly seen in Figure B. In order to bring out the phase relations of these waves in the middle and bottom lines, the space between the first few has been shaded. They are "out of phase" and are therefore coming from the region under the electrode common to the two recorders responsible for the lines showing them. The speed at which the records were taken is shown by the seconds interval in A. The delta waves have a frequency of about three per second. The degree of amplification is shown by the bar between the records: a vertical deflection of the lines of 9 mm. in the original records signifies a change of potential of twenty-millionths of a volt between the electrodes to which the recorder concerned is attached. The delta waves in B have a size from crest to trough of about ten-millionths of a volt.

These minute delta waves then indicate that the area shaded in the brain diagram is in an abnormal condition. There are also normal alpha waves in the top line of B, and these mean that the regions above and behind the shaded part are normal. Many other records were taken from this patient and they all confirmed that the abnormality was limited to the lower central part of the right hemisphere. The only neurological localizing signs in this case were an exaggerated knee-jerk and extensor plantar reflex on the left side. As Professor Short points out, if these guides had been followed, the trephine would have been made much too high and the clot would have been missed, for its centre was actually where the centre of the delta focus had been indicated.

It may be asked : " Is it possible to infer from the record what the nature of the disturbance may be ? " This is difficult to answer. In the previous article on clinical electro-encephalography it was stated that a delta discharge is found in two main pathological groups—expanding lesions and idiopathic epilepsies. Distinction between these two groups can be made in two ways—apart of course from the clinical history, signs and symptoms. Firstly, the site of the discharge in epileptics is most commonly in either the prefrontal or the occipital regions—it is only rarely in the central parts. Secondly, the form of the delta waves in epileptics is more regular than in the other group ; the rhythm is more regular, and there are often, particularly in *petit mal*, absolutely diagnostic complex wave forms. Within the group of space-occupying lesions differential diagnosis is not always possible. In general, the lower the delta frequency, the more acute or malignant the pathological process : also, the greater the size of the waves, the larger the area affected. Occasionally, as a *tour de force* a complete diagnosis has been made by electro-encephalography alone, the site, depth, size and nature of a tumour being foretold. But these attempts have not been uniformly successful. In the tumour group, for example, a slowly growing meningioma in certain sites may give rise to a very slow discharge indistinguishable from that associated with a highly malignant glioblastoma. This is presumably because the solid mass of a meningioma may interfere very seriously with the circulation of blood and cerebro-spinal fluid, thus reducing the nervous elements to a state of extreme dystrophy, while the other benign group of tumours, the astrocytomata, may scarcely interfere at all with the nutrition of the neurones. It is this latter group of tumours which produce the least degree of electro-encephalographic abnormality, and when a tumour has been missed it has nearly always turned out to be an astrocytoma. Therefore, if there are quite unmistakable clinical signs of new growth but the electro-encephalogram is normal or nearly so, the prognosis is good. That an abnormal electro-encephalogram does not always carry a bad outlook is illustrated perfectly by Professor Short's case ; there was nothing in the record to distinguish it from one taken from a case of malignant tumour, but the operation was entirely successful and the relief should be permanent. The apparent ambiguity of the record is explained by the *acuteness* of the lesion. The records were taken only a week after the onset of severe headache and the appearance of serious clinical signs. If there had been no intervention and the patient had survived for a few weeks, it is probable that the electro-encephalogram would gradually have returned to " normal " as the affected cells progressively perished entirely and ceased electrical activity.

This case, then, illustrates three points: firstly, the accuracy of electro-encephalographic location; secondly, the importance of early examination before the nervous tissue has been completely destroyed, and, thirdly, the purely complementary nature of the examination, which can reach its full utility only when correlated with careful clinical observation.

## Figures and Tables

**Figure f1:**